# Empirical Administration of Doxycycline for Rocky Mountain Spotted Fever: A Case Report

**DOI:** 10.7759/cureus.47492

**Published:** 2023-10-22

**Authors:** Proma Dey, Mitara J Mitu, Swarna Chakrabarty, Adrita Nourin Mou, Mohammad F Islam

**Affiliations:** 1 Internal Medicine, Chittagong Medical College, Chattogram, BGD; 2 Internal Medicine, Faridpur Medical College, Dhaka, BGD; 3 Internal Medicine, North East Medical College, Sylhet, BGD; 4 Internal Medicine, Khulna Medical College, Khulna, BGD; 5 Internal Medicine, Cleveland Clinic Akron General, Akron, USA

**Keywords:** empirical antibiotics, tick bite, transaminitis, doxycycline, rocky mountain spotted fever

## Abstract

Rocky Mountain spotted fever (RMSF) is a tick-borne illness that can cause extreme sickness, even death, in otherwise healthy individuals. Sometimes, it is difficult to confirm the diagnosis as the rash often lags behind other symptoms of the illness and may not occur at all. Other symptoms of RMSF are nonspecific, such as fever, headache, and malaise. Besides the confirmatory serology test, antibody titers remain negative in the early phase of the illness. Here, we reported a case of a 21-year-old male who presented with fever, mild headache, body aches, joint pain, dry cough, and characteristic maculopapular rash after visiting a tick-prone area. Doxycycline was started because symptoms and laboratory values heightened our suspicion for the diagnosis of RMSF. His condition improved gradually, and his labs became normal. Our study supports the empirical use of doxycycline in suspected RMSF cases.

## Introduction

Rocky Mountain spotted fever (RMSF) is a tick-borne illness caused by *Rickettsia rickettsii*, an intracellular bacteria, and is transmitted through tick bites. The disease can be fatal. RMSF was first identified in the Rocky Mountain region of the United States and has since been documented in various parts of Canada, Mexico, Central America, and South America (Bolivia, Argentina, Brazil, and Columbia) [1]. In the United States, it is widely prevalent in Arkansas, Missouri, North Carolina, Oklahoma, and Tennessee [2]. These cases are primarily seen in the summertime as the vector of this disease, the *Dermacentor* tick, increases its activity in summer [2]. RMSF manifests with a range of clinical symptoms, including high fever, headache, muscle aches, malaise, arthralgia, nausea, vomiting, and maculopapular rash in the third to fifth days of illness [3]. Other manifestations include hyponatremia, lymphopenia, thrombocytopenia, and coagulopathy, which often occur late in the disease process [4]. Even with the classical presentation, physicians sometimes cannot initiate early treatment because the initial diagnosis must be purely clinical as one-third or more patients with RMSF cannot recall a recent tick bite or recent tick contact [1]. While substantial progress has been made in understanding the epidemiology, pathogenesis, and clinical management of RMSF, challenges remain in accurate diagnosis, public awareness, and tick-borne disease prevention. Prompt administration of appropriate antibiotics, such as doxycycline, is crucial to mitigate the severity of the disease and prevent potential complications [5].

Here, we report a case of a 21-year-old male who recently traveled to Tennessee and presented with fever, body ache, joint pain, and maculopapular rash on the forearms and lower legs as well as torso and neck, and was administered doxycycline early to prevent complications.

## Case presentation

A 21-year-old male with no previous history of illness presented with fever, a mild headache, body aches, joint pain, and a dry cough. These symptoms started four days prior to the presentation. It was only when he noticed a rash that he came to the hospital. He had immigrated from Asia five years prior. There was no recent international travel history. He recently returned from Tennessee five days prior to the presentation. However, he reported that he did not visit the densely forested areas.

On examination, there was a maculopapular, blanching rash over the volar region of the forearm, lower leg, and over his body and neck. He reported no itching or pain over the rash. He had been taking NyQuil since the onset of the fever. Despite this, his temperature was 101°F during admission. On physical examination, his conjunctiva appeared mildly injected. Besides, there was nasal congestion and a sore throat. There was diffuse muscle tenderness and arthralgia. All other systems, including the neurological, cardiovascular, and respiratory systems, were unremarkable.

Based on these findings, measles was suspected, and the patient was placed in isolation. Measles IgM was ordered, which came back negative. Although the patient gave no history of tick bites and denied going to a wooded area, his recent travel to a tick-prone area, characteristic rash and symptoms, and absence of Koplik spot and coryza diverted the diagnosis to RMSF. Moreover, his complete blood count (CBC) on admission showed thrombocytopenia, leukopenia, and hyponatremia, which helped solidify the RMSF diagnosis. His measles IgM was also negative, so measles was ruled out. Considering the late treatment sequence of RMSF, he was started on IV doxycycline 100 mg at 12-hour intervals. His liver enzymes were found to be mildly elevated on admission. His hepatitis panel was ordered. Hepatitis A, B, and C were negative.

**Figure 1:**
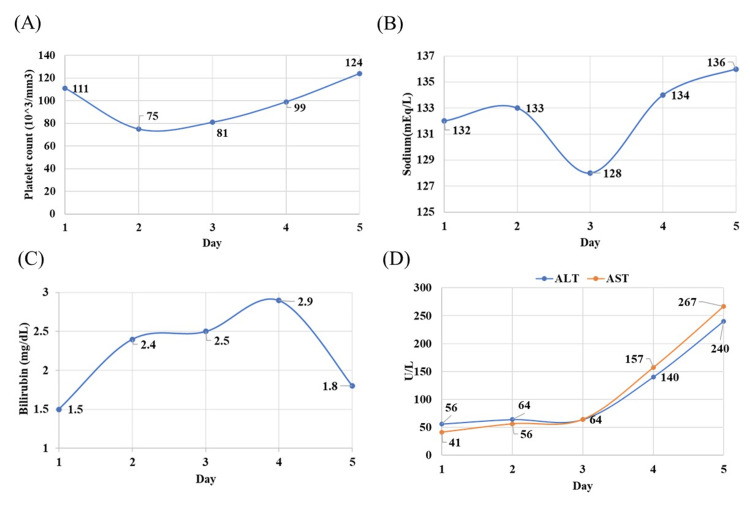
Graphs demonstrating pertinent laboratory value trends during the hospital course: (A) Platelet count (B) Sodium Level (C ) Bilirubin (total) (D) AST and ALT The X-axis shows the days of hospital stay, and the Y-axis shows units of measurement of these laboratory values. AST: Aspartate Transferase; ALT: Alanine Transaminase

An ultrasonogram of the upper abdomen was found unremarkable except for biliary sludge. As shown in Table 1, his liver enzyme (alanine transaminase, aspartate aminotransferase) levels rose daily and peaked on the fifth day of hospitalization. His bilirubin also peaked on the fourth day of hospitalization. His sodium level and platelet count also showed improvement. A graphical representation of his laboratory values is shown in Figure 1. His rash and headache resolved after five days of doxycycline, and the patient was stable. As his situation improved, he was discharged and advised to follow up after a week. 

**Table 1:** Laboratory trend throughout patient’s hospitalization AST: Aspartate Aminotransferase; ALT: Alanine Transaminase

Lab Values	Platelet (10^3/mm3)	Sodium	Total Bilirubin (mg/dL)	ALT (U/L)	AST (U/L)
Day 1	111	132	1.5	56	41
Day 2	75	133	2.4	64	56
Day 3	81	128	2.5	64	64
Day 4	99	134	2.9	140	157
Day 5	124	136	1.8	240	267
Rererence Range	150-400	136-146	0.1-1.0	10-40	12-38

## Discussion

RMSF is a common disease with a high mortality risk, caused by the gram-negative intracellular bacterium *R. rickettsii*. The presentation of RMSF can be varied [6]. The condition can involve different organ systems in our body, like gastrointestinal, renal, neurological, ocular, and musculoskeletal systems [7]. It can cause acute renal failure. Persistent hypotension may lead to prerenal azotemia sometimes. Neurologic manifestations may range from headache, lethargy, photophobia, meningismus, amnesia, ataxia, sensory neuropathy, cranial nerve palsies, confusion, hallucinations, delirium, and coma. Ocular findings include retinal hemorrhages, vasculitis, and vascular occlusion [7]. The classical triad for RMSF is fever, rash, and headache, which can develop in 60-90% of cases, but only 3-18% of cases show these at the initial physician visit [8].

When *R. rickettsii* infects humans, it first invades the endothelial cells, which increases vascular permeability, and those infected cells also increase the release of prostaglandin, platelet-activating factor, and leukotrienes, and thus potentiate a generalized vascular inflammatory reaction [9]. In response to this vascular inflammatory reaction, thrombocytopenia and leukopenia develop, and due to the vascular leaking, the patient develops cerebral edema, the most fatal complication of RMSF [4].

The diagnosis of RMSF depends on the epidemiology, rash pattern, and investigations. Patients can present with typical rickettsial rash involving palm and sole, anemia, leukopenia, thrombocytopenia, and elevated liver enzymes [10]. The confirmatory diagnosis of RMSF is an indirect fluorescent antibody test for IgM and IgG antibodies to* R. rickettsii*. There is a rise in both antibody levels in acute illnesses. IgG is most definitive for RMSF as IgM titers remain elevated for months after the tick exposure. However, the first antibody test during acute illness is often negative as it takes time for them to mount an immune response. For these reasons, empiric treatment with doxycycline is administered to prevent severe complications [4,10].

In this reported case, the patient presented with fever, leukopenia, thrombocytopenia, and elevated liver enzymes with negative hepatic viral serology. Transaminitis can be a predictor of mortality in patients with RMSF [11]. In this case, the patient had typical features of RMSF with opposing other infectious disease panels, including cytomegalovirus, influenza A and B, respiratory syncytial virus, Group A streptococci, HIV, malaria, and coronavirus disease 2019 (COVID-19). 

The standard treatment guideline for RMSF in adults is doxycycline 200 mg/day, given in two divided doses for 7-10 days. Treatment is usually continued until the patient is afebrile for three days. Severe cases may need to be treated by IV administration [12]. The standard treatment guideline in children is 4 mg/kg, given in two divided doses [13]. If RMSF is suspected, empiric treatment should be started immediately, as results of the confirmatory laboratory tests may take a few days to return. Delays in treatment can increase mortality by up to 25% [6]. We followed the standard treatment protocol and empirically treated the patient with IV doxycycline. Empiric treatment in the early phase of the disease helps to prevent severe complications such as seizures, coma, cerebral edema, meningoencephalitis, acute respiratory failure, renal failure, shock, and death [14].

## Conclusions

RMSF, a tick-borne illness, often becomes fatal due to delays in diagnosis and treatment. RMSF should be suspected in patients living or having recent visits to the endemic area and presenting with fever and related symptoms with or without rash. Empiric treatment with doxycycline should be started to reduce mortality and morbidity.

## References

[1] Chen LF, Sexton DJ (2008). What's new in Rocky Mountain spotted fever?. Infect Dis Clin North Am.

[2] Moore SM, McAllister MA, Thomas TO (2023). Rocky Mountain Spotted Fever (RMSF): Epidemiology and statistics. Am J Ophthalmol Case Rep.

[3] Rhodes SD, Teagarden AM, Graner B, Lutfi R, John CC (2020). Brain death secondary to Rocky Mountain spotted fever encephalitis. Case Rep Crit Care.

[4] Zhou C, Woods P, Abouzeid A, Brooks MN (2022). Case report: Rocky Mountain spotted fever with adrenalectomy and a hard-to-find tick. Am J Case Rep.

[5] Wittler RR, Minns GO (2016). An adolescent with fever, rash, and altered mental status. Clin Infect Dis.

[6] Braun DS, Greenberg I, Pagadala M (2021). Rocky Mountain spotted fever masquerading as gastroenteritis: a common but overlooked clinical presentation. Cureus.

[7] Silber JL (1996). Rocky Mountain spotted fever. Clin Dermatol.

[8] Weinberg GA (2007). Rocky Mountain spotted fever. Pediatric Clinical Advisor: Instant Diagnosis and Treatment, 2nd Edn.

[9] Rydkina E, Sahni A, Baggs RB, Silverman DJ, Sahni SK (2006). Infection of human endothelial cells with spotted Fever group rickettsiae stimulates cyclooxygenase 2 expression and release of vasoactive prostaglandins. Infect Immun.

[10] Prasannan A, Ramaswamy P, Anirudhan VK (2017). Rickettsial fever presenting with gangrene: a case series. J Clin Diagn Res.

[11] Zaidi SA, Singer C (2002). Gastrointestinal and hepatic manifestations of tickborne diseases in the United States. Clin Infect Dis.

[12] Nazarian SM, Shaon KY, Schwankhaus JD, Chacko JG, Hudgins PA, Brat DJ (2015). Bilateral optic neuropathy after erythematous rash. Bilateral anterior optic neuropathy due to RMSF. J Neuroophthalmol.

[13] Pace EJ, O'Reilly M (2020). Tickborne diseases: diagnosis and management. Am Fam Physician.

[14] Redford AH, Trost JR, Sibbitt WL Jr, Fangtham M, Emil NS, Singh S, Bankhurst AD (2019). HLA-B27 spondyloarthritis and spotted fever rickettsiosis: case-based review. Rheumatol Int.

